# Personalized Medicine in Otolaryngology: Reflections on a Multidisciplinary Special Issue

**DOI:** 10.3390/jpm16050247

**Published:** 2026-05-01

**Authors:** Mehdi Abouzari

**Affiliations:** Division of Neurotology and Skull Base Surgery, Department of Otolaryngology-Head and Neck Surgery, University of California, Irvine, CA 92697, USA; mabouzar@hs.uci.edu; Tel.: +1-714-509-6096

## 1. Introduction

The practice of medicine has long aspired to treat the individual rather than the disease, yet it has only been in recent decades that the scientific and technological tools required to fulfill this aspiration have begun to mature [1]. In otolaryngology—a specialty that encompasses the remarkably diverse domains of oncology, neurotology, rhinology, laryngology, audiology, and head and neck surgery—the translation of personalized medicine principles into daily clinical practice carries particular relevance. The anatomical complexity, functional intimacy, and profound quality-of-life implications associated with disorders of the ear, nose, throat, and related structures make the “one-size-fits-all” paradigm especially inadequate. It was with this conviction that the present Special Issue was conceived: to gather original research, systematic reviews, consensus statements, and clinical trials that collectively advance our understanding of how individualized approaches can transform outcomes across all subspecialties of otolaryngology.

This closing editorial reflects on the eleven contributions that have shaped this Special Issue, acknowledging the breadth of the methodology, clinical scope, and translational ambition that they represent, and looking ahead to the horizon that their findings illuminate.

## 2. Head and Neck Oncology: From Population Trends to Precision Surgery

Two contributions in this Special Issue directly address the oncological domain, each from a distinct but complementary vantage point. The study by Lin and Hung on the diagnostic gap between clinical and pathological extranodal extension (ENE) in head and neck cancers represents a timely and methodologically rigorous contribution (Contribution 1). Using a five-year nationwide trend analysis from Taiwan, the authors expose a persistent and clinically consequential discordance between radiological or clinical staging and histopathological findings. Given that ENE is among the most powerful prognostic determinants in head and neck squamous cell carcinoma—driving decisions regarding adjuvant chemoradiation, surgical margins, and overall treatment intensity—this gap carries direct implications for individualized treatment planning [2]. The population-level scope of the analysis lends robustness to these findings and underscores the need for improved imaging protocols, intraoperative assessment tools, and perhaps molecular biomarkers that are capable of bridging this diagnostic divide.

Advancing personalized oncological care from a surgical perspective, the work on transoral cordectomy with microelectrodes (TOMES) on an outpatient basis demonstrates the feasibility of leveraging predictive models to tailor the perioperative experience for patients with early-stage laryngeal disease (Contribution 2). By integrating outcome prediction into the decision-making process for outpatient versus inpatient surgical pathways, this study exemplifies how data-driven personalization can simultaneously enhance patient comfort, streamline resource utilization, and maintain oncological safety. The incorporation of microelectrode technology into cordectomy further reflects the broader trajectory of precision surgical instrumentation in the specialty.

## 3. Neurotology and Inner Ear Disorders: Tailoring Rehabilitation and Therapy

The neurotological contributions to this Special Issue are notable both for their clinical relevance and for the heterogeneity of approaches that they employ. The manuscript on personalized vestibular assessment in older patients with cochlear implants addresses one of the more nuanced challenges at the intersection of auditory rehabilitation and balance medicine (Contribution 3). Cochlear implantation is well-established as a transformative intervention for severe-to-profound sensorineural hearing loss, yet its effects on vestibular function—particularly in an aging population that is already predisposed to multifactorial disequilibrium—deserve careful individual characterization [3]. The authors’ call for tailored vestibular assessment protocols in this cohort not only refines candidacy evaluation and preoperative counseling but also informs postoperative rehabilitation strategies, underscoring that successful implantation extends beyond audiological outcomes alone.

Equally innovative is the proposal to repurpose SGLT-2 inhibitors as a novel therapeutic strategy for treatment-resistant Menière’s disease (Contribution 4). Menière’s disease remains one of the most therapeutically challenging conditions in otolaryngology, with a subset of patients failing conventional diuretic, dietary, and intratympanic interventions. By drawing on the well-characterized endolymphatic-decompressing and anti-inflammatory properties of sodium–glucose cotransporter-2 inhibitors—a drug class that was originally developed for glycemic control in type 2 diabetes—this contribution represents a paradigmatic example of pharmacological repurposing guided by pathophysiological reasoning. In an era where personalized pharmacotherapy increasingly incorporates comorbidity profiling and metabolic phenotyping, this proposal invites prospective investigation and may open a novel therapeutic corridor for a historically underserved patient population.

## 4. Rhinology and Sinonasal Disease: Evidence Synthesis and Consensus Building

The rhinological contributions to this Special Issue are united by a commitment to evidence synthesis and the formalization of clinical decision-making in complex scenarios. The systematic review of endoscopic sinus surgery in a frontal sinus inverted papilloma provides a comprehensive appraisal of surgical approaches, recurrence rates, and oncological outcomes in a condition characterized by significant anatomical variability and malignant transformation risk (Contribution 5). The findings reinforce the importance of individualized surgical planning—accounting for tumor origin, Krouse staging, frontal sinus anatomy, and surgeon experience—as determinants of both radicality and functional preservation.

The Young-IfOS consensus on the comprehensive management of cocaine-induced midline destructive lesions (CIMDL) addresses a clinically under-recognized yet growing entity that demands a highly personalized, multidisciplinary approach (Contribution 6). The destructive potential of CIMDL, combined with the psychiatric, social, and immunological complexity of affected patients, renders standardized treatment algorithms insufficient [4]. The consensus framework developed by an international panel of young otolaryngologists provides a valuable decision-support tool while explicitly accommodating patient-specific variables including addiction status, the extent of tissue destruction, immune profile, and psychosocial circumstances. The involvement of a junior faculty consortium also signals a generational commitment to advancing personalized care paradigms in rhinology.

## 5. Audiology and Hearing Rehabilitation: Digital Health and Cognitive Horizons

Two contributions in this Special Issue explore the intersection of audiology with digital health and cognitive medicine, respectively, each reflecting the expanding scope of personalized audiological care. The systematic review and meta-analysis of internet-based therapies for tinnitus synthesizes evidence from randomized controlled trials to evaluate the efficacy of digital cognitive behavioral therapy (CBT), mindfulness-based interventions, and other web-delivered programs in this highly prevalent and quality-of-life-impairing condition (Contribution 7). Tinnitus affects a significant proportion of the adult population, yet access to specialized audiological and psychological care remains inequitable. Internet-delivered therapies hold particular promise in democratizing access to evidence-based intervention, and the granular analysis of effect sizes across outcome domains—tinnitus distress, anxiety, depression, and sleep quality—provides a nuanced basis for matching digital therapeutic modalities to individual patient profiles [5,6].

The study examining the association between hearing aid use and cognitive function in persons with hearing impairment, stratified by cardiovascular risk, addresses one of the most consequential questions in contemporary audiology (Contribution 8). The growing body of evidence linking untreated hearing loss to accelerated cognitive decline has elevated hearing rehabilitation from a quality-of-life intervention to a potential neuroprotective strategy. By stratifying outcomes according to cardiovascular risk—a well-established cognitive comorbidity—the authors contribute important nuance to the personalization of hearing aid prescription and counseling. The findings suggest that the cognitive benefits of amplification may be modulated by vascular health status: a finding with meaningful implications for the integrated management of older patients.

## 6. Laryngology and Voice Medicine: Electrophysiological Phenotyping

The characterization of the normal male and female voice using surface electromyographic (sEMG) parameters represents a foundational contribution to the laryngological component of this Special Issue (Contribution 9). Voice disorders are among the most functionally disabling conditions managed within otolaryngology, affecting professional voice users, patients recovering from laryngeal surgery, and individuals with neurological disease, among others. The establishment of normative sEMG profiles stratified by biological sex provides a critical reference framework for the objective, electrophysiological characterization of dysphonia—complementing acoustic and aerodynamic analyses and paving the way for individualized voice rehabilitation protocols. As artificial intelligence and machine learning tools increasingly enter the diagnostic landscape of laryngology, normative datasets such as those presented here become foundational infrastructure for personalized voice medicine.

## 7. Perioperative Care: Individualized Analgesia After Oropharyngeal Surgery

The randomized, double-blind trial evaluating pain after licorice or sugar–water gargling in patients recovering from oropharyngeal surgery contributes to the underexplored but clinically important domain of personalized perioperative analgesia (Contribution 10). Postoperative pain following tonsillectomy, uvulopalatopharyngoplasty, and related procedures remains a primary determinant of patient satisfaction, recovery duration, and analgesic consumption. The investigation of a simple, low-cost, pharmacologically active gargling intervention as an adjunct to standard analgesia reflects the spirit of personalized care at the bedside—identifying accessible, evidence-based strategies that may benefit specific patient subgroups while minimizing the opioid dependency risk. The rigorous randomized design strengthens the evidentiary value of this contribution.

## 8. Methodology and Reporting Standards: The Delphi Consensus in Otolaryngology

Underpinning all clinical and translational endeavors in personalized medicine is the quality of the evidence and consensus processes that guide them. The systematic review assessing the reliability and reporting completeness of Delphi consensus studies in otolaryngology addresses a methodological dimension that is of critical importance (Contribution 11). Delphi methodology is increasingly deployed in our specialty to formalize expert agreement in areas where randomized evidence is sparse—precisely the conditions that often characterize emerging personalized medicine interventions. The identification of heterogeneity in reporting standards and the proposal of quality benchmarks represent a significant contribution to the methodological rigor of the field, ensuring that future consensus statements—such as those included in this Special Issue—are held to transparent and reproducible standards.

## 9. Synthesis and Outlook

Taken together, the eleven papers assembled in this Special Issue articulate a compelling and multi-dimensional vision of personalized medicine in otolaryngology. Several cross-cutting themes emerge from this collection. First, the importance of biomarker-driven and data-informed stratification is evident across contributions ranging from ENE assessment in head and neck cancer to cardiovascular risk stratification in audiological rehabilitation. The trajectory of the field points unmistakably toward the integration of molecular, genetic, electrophysiological, and digital health data streams into routine clinical workflows. Second, the therapeutic potential of drug repurposing, digital health platforms, and minimally invasive surgical innovation underscores that personalization is not synonymous with complexity or cost. Many of the most promising individualized interventions described in this Special Issue are accessible, scalable, and grounded in established biological rationale. Third, the methodological diversity of contributions—encompassing nationwide epidemiological analyses, randomized controlled trials, systematic reviews, meta-analyses, consensus processes, and basic science characterization studies—reflects the pluralistic evidentiary foundation that personalized medicine requires. No single methodology suffices; rather, it is the convergence of complementary approaches that advances understanding. Fourth, the recurring emphasis on quality of life—from vestibular rehabilitation following cochlear implantation, to tinnitus management, to postoperative pain control—affirms that personalized medicine in otolaryngology is not exclusively about survival or disease eradication, but it is about the restoration and preservation of the full spectrum of human sensory and communicative function.

Looking ahead, the field will be shaped by advances in genomics, proteomics, and microbiomics as applied to head and neck oncology and chronic sinonasal disease; by the maturation of gene and stem cell therapies for hereditary hearing loss and laryngotracheal disorders; and by the integration of artificial intelligence into diagnostic imaging, audiological assessment, and surgical planning. The contributions in this Special Issue represent not an endpoint but a foundation—a demonstration of what rigorous, clinically motivated, and methodologically diverse scholarship can achieve in advancing individualized care for patients across the full breadth of our specialty.

I am deeply grateful to all the authors, reviewers, and editorial colleagues whose intellectual generosity and scientific rigor made this Special Issue possible. It is my sincere hope that the work presented here will serve as both a reference and an inspiration for the next generation of clinicians and researchers who are committed to the promise of personalized medicine in otolaryngology.
